# *Schisandra chinensis* alleviates dextran sulfate sodium-induced colitis in association with remodeling of a *Parabacteroides goldsteinii*-related leucine–isovalerate metabolic axis and attenuation of mTOR/HIF-1α/IL-17 signaling

**DOI:** 10.3389/fmicb.2026.1941347

**Published:** 2026-09-09

**Authors:** Jiantao Song, Hongfei Kan, Huaijie Jin, Lin Yue, Zhouyi Qian, Bo Wang, Zongling Xia, Yanan Zhou, Jiuba Zhang

**Affiliations:** 1 Department of Pharmacy, The First People's Hospital of Changzhou, Changzhou, China; 2 College of Pharmacy, Nanjing University of Chinese Medicine, Nanjing, China; 3 Department of Neurosurgery, Shanghai Changhai Hospital, Naval Medical University, Shanghai, China

**Keywords:** leucine–isovalerate axis, mTOR/HIF-1α/IL-17 signaling, *Parabacteroides goldsteinii*, *Schisandra chinensis*, ulcerative colitis

## Abstract

Ulcerative colitis (UC) involves epithelial injury, gut microbial dysbiosis, disturbed microbial metabolism, and mucosal inflammation. This study evaluated the protective effect of *Schisandra chinensis* (SC) against dextran sulfate sodium (DSS)-induced UC and investigated the underlying microbiota–metabolite–host signaling mechanism. UPLC-Q-TOF-MS/MS identified 38 constituents in SC, predominantly lignans, organic and phenolic acids, and terpenoids. SC reduced body weight loss, disease activity, colon shortening, mucosal and histological injury, and pro-inflammatory cytokine levels, while partially restoring claudin-1 and occludin expression. Metagenomic analysis showed that SC restructured the DSS-disrupted microbiota, enriched *Parabacteroides goldsteinii*, and increased the predicted abundance of pathways related to valine, leucine, and isoleucine degradation. Untargeted metabolomics and targeted fatty acid analysis showed lower fecal leucine and higher isovalerate levels after SC treatment. Whole-colon transcriptomic and western blot analyses further demonstrated that SC suppressed the mTOR/HIF-1α/STAT3/IL-17 inflammatory program in colonic tissue. Antibiotic pretreatment weakened several protective effects of SC, whereas *P. goldsteinii* or sodium isovalerate administration partially recapitulated its anti-inflammatory and barrier-restorative effects. Conversely, the mTOR activator MHY1485 attenuated SC-mediated protection. These findings indicate that the protective effects of SC are associated with remodeling of a *P. goldsteinii*-related leucine–isovalerate metabolic network and attenuation of colonic mTOR/HIF-1α/STAT3/IL-17 signaling. This study provides a microbiota-centered mechanistic basis for the therapeutic potential of SC in UC.

## 1 Introduction

Ulcerative colitis (UC) is a chronic, relapsing inflammatory bowel disease that primarily affects the colonic and rectal mucosa. Its major clinical manifestations include diarrhea, abdominal pain, bloody or mucus-containing stools, and systemic symptoms of varying severity. In recent years, the number of patients living with inflammatory bowel disease has continued to increase in traditionally high-incidence regions, while incidence has risen markedly in rapidly industrializing countries and regions. Inflammatory bowel disease has therefore become a major global gastrointestinal disease burden (Hracs et al., 2025; Kaplan and Windsor, 2021; Kobayashi et al., 2020). Current treatments, including 5-aminosalicylates, corticosteroids, immunosuppressants, biologics, and targeted small molecules, can improve clinical symptoms and mucosal inflammation in some patients. Nevertheless, primary nonresponse, secondary loss of response, relapse, infection risk, and the high cost of long-term treatment remain substantial challenges (Kobayashi et al., 2020; Stalgis et al., 2021). Interventions that simultaneously restore the mucosal barrier, intestinal ecology, and dysregulated immune responses are therefore needed.

The UC is not driven by a single factor. Instead, it arises from interactions among genetic susceptibility, environmental exposures, the gut microbiota, epithelial barrier dysfunction, and mucosal immune imbalance (Chang, 2020; Kobayashi et al., 2020). The intestinal mucosal barrier is the principal defense that restricts luminal antigens, microorganisms, and microbial products from entering the lamina propria. Occludin, claudins, and zonula occludens proteins maintain epithelial tight junctions. Barrier disruption increases intestinal permeability and permits sustained entry of bacterial components and inflammatory signals into mucosal tissues, thereby creating a self-reinforcing cycle of barrier injury, immune activation, and further barrier deterioration (Mehandru and Colombel, 2021; Odenwald and Turner, 2017). Patients with UC also commonly exhibit reduced microbial diversity, unstable community composition, and extensive remodeling of microbial metabolic functions. Longitudinal multi-omics studies have shown that active inflammatory bowel disease is associated not only with compositional changes in the microbiota, but also with abnormalities in short-chain fatty acids, bile acids, tryptophan derivatives, amino acids, and lipid mediators (Franzosa et al., 2019; Lavelle and Sokol, 2020; Lloyd-Price et al., 2019; Ning et al., 2023). Defining microbial metabolic functions and their effects on host signaling may therefore reveal functional links between dysbiosis and disease more effectively than describing taxonomic abundance alone.

Branched-chain amino acids (BCAAs) serve as both nutritional substrates and important metabolic signals. Leucine activates mechanistic target of rapamycin complex 1 (mTORC1) through nutrient-sensing systems such as the Sestrin2–GATOR2 pathway (Saxton et al., 2016; Wolfson et al., 2016). mTOR signaling subsequently regulates glycolysis, cell growth, immune-cell differentiation, and inflammatory cytokine production (Liu and Sabatini, 2020). Hypoxia-inducible factor-1α (HIF-1α) links the inflammatory microenvironment to cellular metabolism. It promotes Th17-associated metabolic programs and RORγt expression and contributes to regulation of the Th17/Treg balance (Dang et al., 2011; Shi et al., 2011). Persistent activation of the STAT3–RORγt–IL-17 program is closely associated with intestinal mucosal inflammation, while mTOR and HIF-1α may act as key nodes through which nutrient signals drive Th17 responses (Kumar et al., 2021; Stockinger and Omenetti, 2017). Intestinal bacteria can also ferment leucine to produce branched-chain fatty acids, including isovalerate. In contrast to acetate, propionate, and butyrate, however, the effects of isovalerate on the intestinal barrier and mucosal immunity have received limited systematic attention (Diether and Willing, 2019; Neis et al., 2015; Portune et al., 2016). A recent study showed that leucine-derived isovalerate enhances intestinal epithelial barrier function and exhibits partial histone deacetylase-inhibitory activity, providing direct evidence that isovalerate can function as a bioactive microbial metabolite (Beaumont et al., 2026).

*Schisandra chinensis* (SC), a member of the Magnoliaceae family, is used medicinally as its dried ripe fruit. It contains lignans, polysaccharides, organic acids, volatile constituents, triterpenoids, and other compounds (Rybnikář et al., 2024; Skalski et al., 2025). Previous studies have demonstrated anti-inflammatory, antioxidant, hepatoprotective, and microbiota-modulating effects of SC and its lignan and polysaccharide fractions (Rybnikář et al., 2024; Skalski et al., 2025). In dextran sulfate sodium (DSS)-induced colitis models, SC extracts or polysaccharides reduced tissue injury, regulated inflammatory cytokines, restored tight junction proteins, and improved microbial composition and metabolism (Bian et al., 2022; Guo et al., 2024; Su et al., 2020; Wang et al., 2023; Zhang et al., 2023). Most available studies, however, have focused on TLR4/NF-κB signaling, the NLRP3 inflammasome, or global changes in community structure. Whether SC enriches specific functional bacteria, alters BCAA-derived metabolic signals, and thereby regulates host nutrient-sensing pathways remains unclear.

*Parabacteroides goldsteinii* is an obligately anaerobic intestinal bacterium with potential probiotic properties. Previous work showed that *P. goldsteinii* improves obesity-associated metabolic dysfunction and intestinal barrier integrity and can be enriched by several polysaccharide interventions (Wu et al., 2019). More recent studies indicate that *P. goldsteinii* can ameliorate experimental colitis through mechanisms involving PI3K–Akt signaling, bile acid metabolism, or BCAA-derived metabolites (He et al., 2025; Li et al., 2024; Qin et al., 2025). Its metabolic output, however, may vary with strain identity and nutritional environment. It is not known whether the enrichment of *P. goldsteinii* induced by SC is associated with remodeling of leucine–isovalerate metabolism and mTOR-related inflammatory signaling.

To address these questions, we used a DSS-induced mouse model of colitis and combined UPLC-Q-TOF-MS/MS, metagenomic sequencing, untargeted metabolomics, targeted fatty acid quantification, and colonic transcriptomics. We systematically evaluated the protective efficacy of SC and its effects on the microbiota–metabolite–host signaling network. Microbiota depletion, *P. goldsteinii* supplementation, sodium isovalerate administration, and reversal with an mTOR activator were then used to evaluate the functional contributions of the gut microbiota, the candidate bacterium, isovalerate, and mTOR signaling to the protective effects of SC against colitis.

## 2 Materials and methods

### 2.1 Preparation of the *Schisandra chinensis* decoction

Dried SC fruits (200 g) were decocted twice with 1,200 and 800 mL of water, respectively, for 1 h each. The two extracts were combined and concentrated under reduced pressure to a final concentration equivalent to 1.6 g of crude drug per milliliter. The concentrated decoction was stored at −20 °C, thawed at 4 °C before use, and diluted with water to the required dosing concentrations.

### 2.2 UPLC-Q-TOF-MS/MS analysis of SC

An aliquot of SC extract (10 μL) was mixed with 490 μL of methanol, vortexed for 5 min, and centrifuged at 12000 × g for 10 min at 4 °C. The supernatant was passed through a 0.22-μm membrane filter before UPLC-Q-TOF-MS/MS analysis. Chromatographic separation was performed on an Agilent ZORBAX Extend-C18 column (2.1 × 100 mm, 1.8 μm). Mobile phase A was water containing 0.1% formic acid, and mobile phase B was acetonitrile. The flow rate was 0.3 mL/min, the column temperature was 25 °C, and the injection volume was 1 μL. The gradient was as follows: 0–25 min, 5–95% B; 25–27 min, 95% B; 27–29 min, 95–5% B; and 29–30 min, 5% B. Mass spectrometric data were acquired using an electrospray ionization source in both positive- and negative-ion modes. The spray voltages were 5,500 and −4,500 V, the declustering potentials were 60 and −60 V, and the collision-cell exit potentials were 10 and −10 V in positive- and negative-ion modes, respectively. The nebulizer gas, auxiliary heating gas, and curtain gas pressures were set to 55, 55, and 35 psi, respectively, and the ion-source temperature was 550 °C. The TOF-MS scan range was m/z 100–1,500, and the TOF-MS/MS scan range was m/z 50–1,000. The collision energy was 40 V with a collision-energy spread of 10 V.

### 2.3 Animals

Male C57BL/6 mice, 6–8 weeks of age and weighing 18–22 g, were purchased from Jiangsu GemPharmatech Co., Ltd. (license no. SCXK [SU]-2023–0009). The mice were housed under specific-pathogen-free animal facility at 22 ± 2 °C and 50–60% relative humidity under a 12-h light/12-h dark cycle, with free access to standard chow and water. After a 7-day of acclimatization, the mice were allocated to experimental groups using a random-number method. All animal procedures were approved by the Experimental Animal Ethics Committee of Nanjing University of Chinese Medicine (approval no. 202311A048).

### 2.4 Experimental design

#### 2.4.1 Experiment 1: evaluation of the protective effects of SC against DSS-induced ulcerative colitis

Mice were randomly assigned to five groups (n = 6 per group): Control, DSS, Mesalazine, SC low-dose (SCL), and SC high-dose (SCH). Mesalazine was administered at 100 mg/kg, and SC was administered at 400 or 1,600 mg/kg in the low- and high-dose groups, respectively. The SC doses were selected with reference to the upper recommended daily dose of 6 g for adults specified in the Chinese Pharmacopoeia (Chinese Pharmacopoeia Commission, 2025). Assuming a 70-kg adult and applying a human-to-mouse body surface area conversion factor of 9.1, the mouse-equivalent dose was calculated as (6,000 mg/70 kg) × 9.1 = 780 mg/kg and rounded to 800 mg/kg as the reference dose. To evaluate a dose range around this clinically equivalent level while limiting animal use, 400 mg/kg (0.5-fold) and 1,600 mg/kg (2-fold) were selected as the low and high doses, respectively. Except for the Control group, mice received 2.5% DSS in drinking water for 7 consecutive days to induce acute colitis (Wirtz et al., 2017; Yang and Merlin, 2024). Control mice received regular drinking water during the same period. Mesalazine and SC were administered once daily by oral gavage at 5 mL/kg. Control and DSS mice received an equal volume of physiological saline. Body weight, stool consistency, and fecal occult blood were recorded daily.

#### 2.4.2 Experiment 2: microbiota depletion and *Parabacteroides goldsteinii* supplementation

Mice were randomly assigned to Control, DSS, SC, antibiotic cocktail plus SC (ABX + SC), and *Parabacteroides goldsteinii* groups (*n* = 6 per group). All groups except the Control group received 2.5% DSS to induce colitis. Mice in the SC and ABX + SC groups received SC at 1600 mg/kg once daily by gavage. Before DSS exposure, mice in the ABX + SC and *P. goldsteinii* groups were gavaged with 200 μL of an antibiotic cocktail once daily for 7 days. The ABX contained vancomycin (100 mg/kg), neomycin (200 mg/kg), metronidazole (200 mg/kg), and ampicillin (200 mg/kg) (Kennedy et al., 2018). A 1-day washout period was allowed after antibiotic treatment, after which DSS and SC were administered as described above. Fecal samples were collected before and after antibiotic treatment to assess microbiota depletion.

*Parabacteroides goldsteinii* JCM 13446ᵀ was inoculated into Gifu anaerobic medium and cultured at 37 °C under strictly anaerobic conditions for 24 h (Zhao et al., 2025). The culture was centrifuged at 6,000 × *g* for 10 min, and the bacterial pellet was resuspended in anaerobic PBS. Viable counts were determined by plate counting, and the suspension concentration was adjusted accordingly. Mice in the *P. goldsteinii* group received 2 × 10^8^ CFU of viable bacteria in 200 μL by daily oral gavage for 7 days. Control, DSS, and the other intervention groups received an equal volume of vehicle.

#### 2.4.3 Experiment 3: sodium isovalerate supplementation and mTOR activation

Mice were randomly assigned to Control, DSS, SC, sodium isovalerate, and SC + MHY1485 groups (*n* = 6 per group). Except for the Control group, all mice received 2.5% DSS to induce acute colitis. The SC group received SC at 1,600 mg/kg, the sodium isovalerate group received sodium isovalerate at 100 mg/kg, and the SC + MHY1485 group received SC at 1600 mg/kg together with MHY1485 at 2.5 mg/kg. SC and sodium isovalerate were administered once daily by oral gavage. MHY1485 was administered once daily by intraperitoneal injection.

### 2.5 Disease activity index assessment

Body weight, stool consistency, and fecal occult blood were recorded at a fixed time each day. The disease activity index (DAI) was calculated as the mean of the scores for body weight loss, stool consistency, and fecal occult blood. Body weight loss was scored as follows: <1%, 0; 1–5%, 1; 5–10%, 2; 10–20%, 3; and >20%, 4. Stool consistency was scored as follows: normally formed stool, 0; soft stool, 2; and watery diarrhea, 4. Fecal occult blood was scored as follows: negative fecal occult blood, 0; positive fecal occult blood, 2; and grossly visible blood, 4.

### 2.6 Sample collection and colon assessment

At the end of the experiment, mice were anesthetized with isoflurane inhalation anesthesia (3% for induction and 1–2% for maintenance). Blood samples were collected from the orbital venous plexus, followed by euthanasia through cervical dislocation. Blood samples were allowed to stand at room temperature for 2 h and then centrifuged at 12,000 × *g* for 10 min at 4 °C. The resulting serum samples were collected and stored at −80 °C. After euthanasia, the colon was excised from the distal cecum to the anus and photographed. The colonic mucosal damage index (CMDI) was scored as follows: normal mucosa, 0; mild hyperemia without ulceration, 1; marked hyperemia with a small number of punctate ulcers, 2; ulceration <1 cm in length, 3; and ulceration >1 cm or extensive mucosal injury, 4.

A distal colon segment of approximately 0.5 cm was fixed in 4% paraformaldehyde for histopathology, immunofluorescence, and immunohistochemistry. The remaining colonic tissue was snap-frozen in liquid nitrogen and stored at −80 °C for enzyme-linked immunosorbent assay (ELISA), western blotting, and transcriptomic analysis. Fresh fecal samples were immediately transferred to sterile cryovials, snap-frozen in liquid nitrogen, and stored at −80 °C for metagenomic sequencing and fatty acid analysis.

### 2.7 Histopathological examination

Fixed colonic tissues were dehydrated through a graded ethanol series, cleared in xylene, embedded in paraffin, and cut into serial 4 μm sections. After deparaffinization and rehydration, the sections were stained with hematoxylin and eosin and examined by light microscopy. Histological injury was assessed according to inflammatory cell infiltration, mucosal and crypt damage, epithelial integrity, and the extent of ulceration. Scores were assigned as follows: 0, normal colonic mucosa without appreciable thickening of the lamina propria; 1, mild thickening of the lamina propria without evident inflammatory cell infiltration; 2, moderate thickening of the lamina propria accompanied by inflammatory cell infiltration; 3, pronounced thickening of the lamina propria with extensive inflammatory cell infiltration around the crypts or within the mucosa; and 4, transmural inflammation involving the entire bowel wall.

### 2.8 Measurement of inflammatory cytokines

Colonic tissue (50 mg) was mixed with 450 μL of ice-cold PBS and homogenized thoroughly on ice. The homogenates were centrifuged at 12,000 × *g* for 10 min at 4 °C, and the supernatants were collected for analysis. The concentrations of TNF-α, IL-1β, IL-6, IL-4, and IL-10 were determined using mouse-specific ELISA kits according to the manufacturers’ protocols. Standards and tissue homogenate supernatants were added to the microplates and sequentially incubated with the corresponding detection reagents, substrate solution, and stop solution. Absorbance was measured at 450 nm using a microplate reader, and cytokine concentrations were calculated from the corresponding standard curves.

### 2.9 Immunofluorescence staining

Paraffin-embedded colon sections were deparaffinized in xylene, rehydrated through a graded ethanol series, and rinsed with distilled water. Heat-induced antigen retrieval was performed in sodium citrate buffer (10 mmol/L, pH 6.0) at 95–100 °C for 15 min. After cooling to room temperature, the sections were washed three times with PBS and blocked with 3% bovine serum albumin for 30 min at room temperature to reduce nonspecific binding. The sections were then incubated overnight at 4 °C with primary antibodies against occludin (1:500) or claudin-1 (1:1000). After three washes with PBS, the sections were incubated with the appropriate fluorophore-conjugated secondary antibodies for 60 min at room temperature in the dark. Nuclei were counterstained with DAPI for 10 min, and the sections were mounted using an antifade mounting medium. Fluorescence images were acquired by confocal microscopy using identical laser intensity, exposure time, gain, and magnification settings for all experimental groups.

### 2.10 Immunohistochemical staining

Paraffin-embedded colon sections were deparaffinized in xylene, rehydrated through a graded ethanol series, and subjected to heat-induced antigen retrieval in sodium citrate buffer (10 mmol/L, pH 6.0) at 95–100 °C for 15 min. After cooling to room temperature, the sections were washed three times with PBS. Endogenous peroxidase activity was blocked by incubation with 3% hydrogen peroxide for 10 min, followed by three washes with PBS. Nonspecific binding was blocked with 3% bovine serum albumin for 30 min at room temperature. The sections were incubated overnight at 4 °C with primary antibodies against p-mTOR (1:2000), HIF-1α (1:1000), or IL-17A (1:1000). After three washes with PBS, the sections were incubated with an HRP-conjugated secondary antibody (1:2500) for 60 min at room temperature. Immunoreactivity was visualized using 3,3′-diaminobenzidine, and color development was monitored microscopically and terminated by rinsing the sections with distilled water. Nuclei were counterstained with hematoxylin. The sections were subsequently dehydrated through a graded ethanol series, cleared in xylene, and mounted with neutral resin. Images were acquired using a light microscope under identical illumination, magnification, and acquisition settings for all experimental groups.

### 2.11 Western blot analysis

Colonic tissues were homogenized on ice in RIPA lysis buffer containing 1% protease inhibitor and 1% phosphatase inhibitor. Lysates were centrifuged at 12,000 × *g* for 10 min at 4 °C, and the supernatants were collected. Total protein concentrations were determined using a bicinchoninic acid assay. Equal amounts of protein were separated by SDS-PAGE and transferred to PVDF membranes. Membranes were blocked with 5% bovine serum albumin for 1 h at room temperature and incubated overnight at 4 °C with rabbit primary antibodies against occludin (1:2500), claudin-1 (1:5000), mTOR (1:5000), p-mTOR (1:1000), STAT3 (1:5000), p-STAT3 (1:5000), HIF-1α (1:10000), RORγt (1:5000), IL-17A (1:5000), and GAPDH (1:7500). After three washes with TBST, membranes were incubated with HRP-conjugated goat anti-rabbit IgG (1:10000) for 2 h at room temperature. Protein bands were visualized by enhanced chemiluminescence and captured with a chemiluminescence imaging system, and band intensities were quantified using ImageJ.

### 2.12 Metagenomic sequencing

Fresh fecal samples were collected in sterile cryovials and stored at −80 °C. Total microbial DNA was extracted using a fecal DNA extraction kit. DNA concentration and purity were measured using NanoDrop and Qubit instruments, and DNA integrity was evaluated by agarose gel electrophoresis. Qualified DNA samples were used to construct metagenomic libraries, which were subjected to paired-end sequencing on an Illumina NovaSeq X Plus platform. Predicted genes were clustered with CD-HIT to generate a non-redundant gene catalog (Li and Godzik, 2006). Amino acid sequences from the non-redundant catalog were aligned against the NR database using DIAMOND to obtain taxonomic assignments and abundance profiles. Functional annotation was performed by comparison with the Kyoto Encyclopedia of Genes and Genomes (KEGG) database (Buchfink et al., 2021; Kanehisa et al., 2016).

### 2.13 Untargeted metabolomics

Fecal samples (50 mg) were extracted with 600 μL of methanol–water (4:1, v/v) containing 2-chloro-L-phenylalanine as an internal standard. Samples were homogenized at 60 Hz for 120 s at 4 °C and then sonicated in an ice bath for 10 min. After centrifugation at 12000 × g for 10 min at 4 °C, the supernatants were passed through 0.22-μm membrane filters for LC–MS analysis. Equal aliquots from all samples were pooled to prepare quality control samples.

Chromatographic separation was performed using a Thermo Ultimate 3,000 ultrahigh-performance liquid chromatography system equipped with an ACQUITY UPLC HSS T3 column (2.1 × 100 mm, 1.8 μm). The flow rate was 0.3 mL/min, the column temperature was 40 °C, and the injection volume was 2 μL. In positive-ion mode, mobile phase A was water containing 0.1% formic acid and mobile phase B was acetonitrile containing 0.1% formic acid. In negative-ion mode, mobile phase C was 5 mmol/L aqueous ammonium formate and mobile phase D was acetonitrile. The same gradient was used in both ionization modes: 0–1 min, 10% organic phase; 1–7.5 min, 10–98%; 7.5–10 min, 98%; 10–10.1 min, 98–10%; and 10.1–12 min, 10%.

Mass spectrometry was performed on a Thermo Q Exactive high-resolution mass spectrometer equipped with an electrospray ionization source. Data were acquired in both positive- and negative-ion modes. The spray voltages were 3.50 and −2.50 kV, respectively. The sheath gas and auxiliary gas were set to 40 and 10 arbitrary units, and the capillary temperature was 325 °C. Full-scan spectra were acquired over m/z 81–1,000 at a resolution of 70,000. Tandem mass spectra were acquired by higher-energy collisional dissociation at 30 eV and a resolution of 17,500.

### 2.14 Quantification of fecal fatty acids

Fresh fecal samples (50 mg) were mixed with 500 μL of ultrapure water and 100 mg of glass beads and homogenized for 1 min. The samples were centrifuged at 12000 × g for 10 min at 4 °C. An aliquot of supernatant (200 μL) was mixed with 100 μL of 15% phosphoric acid, 20 μL of 4-methylvaleric acid internal standard solution (375 μg/mL), and 280 μL of diethyl ether. After vortexing for 1 min and centrifugation, the upper ether layer was collected for GC–MS analysis.

GC–MS was performed using a Thermo TRACE 1300 gas chromatograph coupled to an ISQ 7000 mass spectrometer and fitted with an Agilent HP-INNOWAX capillary column (30 m × 0.25 mm × 0.25 μm). The injection volume was 1 μL, the split ratio was 10:1, and helium was used as the carrier gas at 1.0 mL/min. The inlet, ion-source, and transfer-line temperatures were 250, 300, and 250 °C, respectively. The oven temperature was increased from 90 to 120 °C at 10 °C/min, then to 150 °C at 5 °C/min, and finally to 250 °C at 25 °C/min, where it was held for 2 min. The mass spectrometer was operated using electron-impact ionization in selected-ion monitoring mode at 70 eV.

### 2.15 RNA sequencing

Total RNA was extracted from colonic tissue using TRIzol reagent. RNA concentration and purity were assessed with a NanoDrop 2000 spectrophotometer, while integrity was evaluated by agarose gel electrophoresis and an Agilent Bioanalyzer. Paired-end libraries were sequenced on an Illumina NovaSeq X Plus platform. After quality control, clean reads were aligned to the mouse reference genome. Gene expression was expressed as transcripts per million, whereas raw read counts were used for differential expression analysis. Differentially expressed genes were identified using DESeq2 with thresholds of adjusted *p* < 0.05 and |log_2_ fold change| > 2 (Love et al., 2014). Gene set enrichment analysis was performed using the ranked list of all genes, and normalized enrichment scores, nominal *p* values, and false discovery rate q values were calculated (Subramanian et al., 2005).

### 2.16 Quantitative real-time PCR

Total RNA was extracted from colonic tissues using TRIzol reagent according to the manufacturer’s instructions. RNA concentration and purity were assessed by measuring absorbance at 260 and 280 nm. Equal amounts of total RNA were reverse-transcribed into complementary DNA (cDNA), which was subsequently used as the template for quantitative real-time PCR (qRT-PCR). qRT-PCR was performed using a real-time fluorescence quantitative PCR system (LT6276, Gene Company) with SYBR Green Master Mix in a final reaction volume of 20 μL. The amplification program consisted of an initial denaturation at 95 °C for 30 s, followed by 40 cycles of denaturation at 95 °C for 5 s and annealing/extension at 60 °C for 30 s. A melting-curve analysis was subsequently performed to confirm the specificity of amplification. The primer sequences were as follows: RORC, forward 5′-CTTCACCCCACCTCCACTG-3′ and reverse 5′-CTGAGAACTTGGCTCCCTGTC-3′; IL-17A, forward 5′-GCTCCAGAAGGCCCTCAGA-3′ and reverse 5′-CTTTCCCTCCGCATTGACA-3′; Hif1a, forward 5′-GCCTTAACCTGTCTGCCACTT-3′ and reverse 5′-TTCGCTTCCTCTGAGCATTCTG-3′; m-TOR, forward 5′-ACCGGCACACATTTGAAGAAG-3′ and reverse 5′-CTCGTTGAGGATCAGCAAGG-3′; and GAPDH, forward 5′-TGCACCACCAACTGCTTAG-3′ and reverse 5′-AGAGGCAGGGATGATGTTC-3′.

### 2.17 Statistical analysis

Statistical analyses were performed using GraphPad Prism. Data are presented as the mean ± standard error of the mean. Normality was assessed using the Shapiro–Wilk test, and homogeneity of variance was evaluated using the Brown–Forsythe or Levene test.

Comparisons between two groups were performed using two-tailed unpaired Student’s t-tests. Normally distributed data with homogeneous variances across multiple groups were analyzed by one-way analysis of variance followed by Tukey’s multiple-comparison test. Non-normally distributed data were analyzed using the Kruskal–Wallis test followed by Dunn’s multiple-comparison test. All tests were two-sided, and *p* < 0.05 was considered statistically significant.

## 3 Results

### 3.1 UPLC-Q-TOF-MS/MS profiling defines the chemical composition of SC decoction

**Figure 1:**
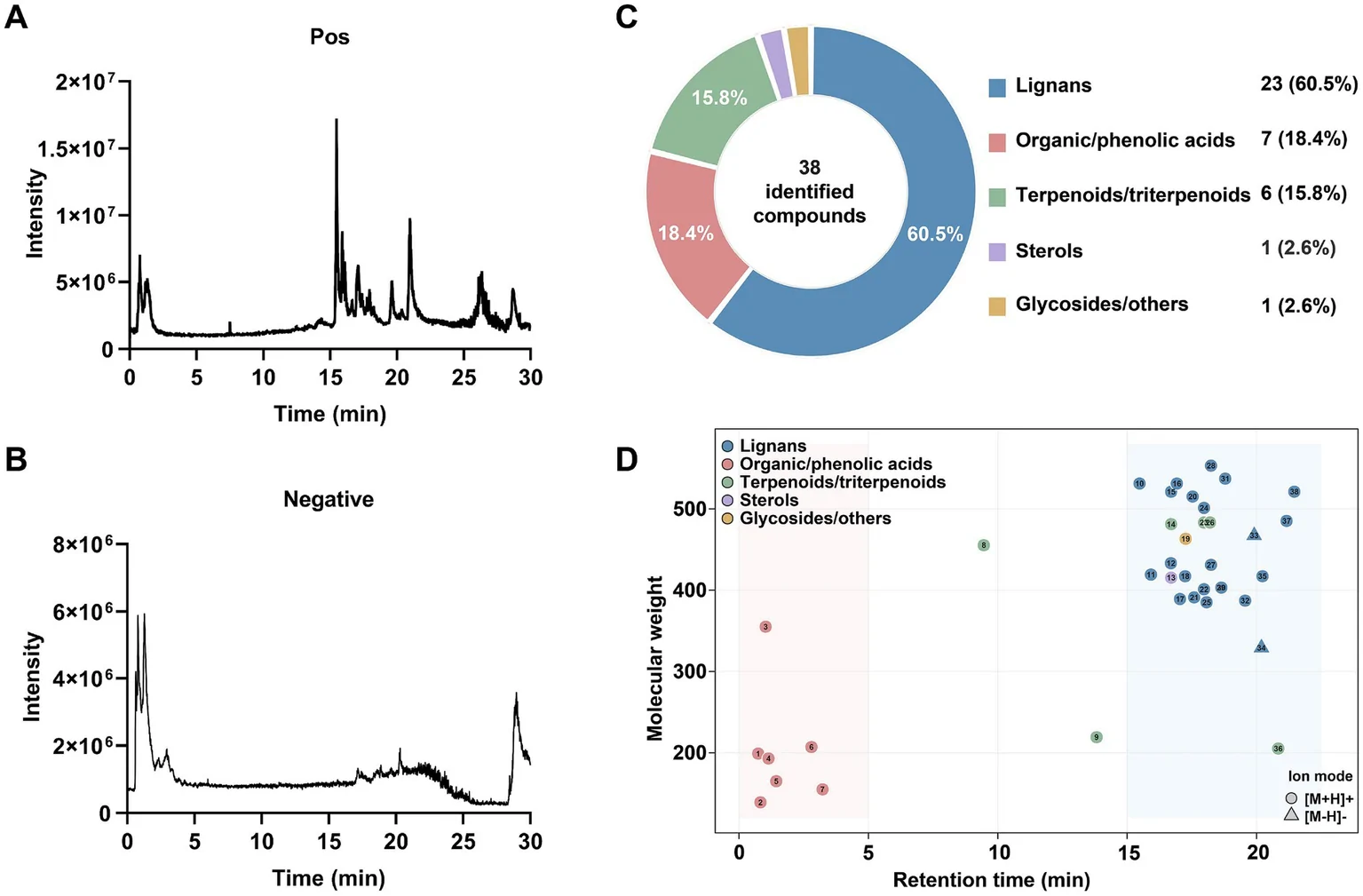
Chemical profiling of the *Schisandra chinensis* (SC) decoction by UPLC-Q-TOF-MS/MS. **(A,B)** Total-ion chromatograms acquired in positive-ion mode **(A)** and negative-ion mode **(B)**. **(C)** Classification of the 38 tentatively identified compounds. **(D)** Distribution of identified compounds according to retention time and molecular weight. Compound numbers correspond to Supplementary Table S1.

To characterize the principal chemical constituents of the SC decoction, UPLC-Q-TOF-MS/MS was performed in both positive- and negative-ion modes. The total-ion chromatograms contained numerous characteristic peaks throughout the 0–30-min acquisition window, indicating that the decoction comprised multiple classes of compounds with different polarities and retention behaviors. The overall response was stronger in positive-ion mode, with several high-intensity peaks between approximately 15 and 22 min. In negative-ion mode, prominent signals were observed in the early-eluting region, together with several peaks in the later portion of the chromatogram (Figures 1A,B). The complementary ionization patterns indicated that the decoction retained water-soluble organic and phenolic acids as well as lignans and terpenoids with longer retention times.

On the basis of retention time, accurate mass, ion-adduct form, mass error, and MS/MS fragmentation, 38 compounds were tentatively identified in the decoction. Of these, 36 were detected predominantly in positive-ion mode and two in negative-ion mode (Supplementary Table S1). Lignans were the most abundant chemical class, with 23 compounds representing 60.5% of all identified constituents. Seven organic or phenolic acids accounted for 18.4%, and six terpenoid or triterpenoid compounds accounted for 15.8%. One sterol and one glycoside or other compound each represented 2.6% of the total (Figure 1C).

Polar compounds identified in the early-eluting region included syringic acid, 4-hydroxybenzoic acid, chlorogenic acid, citric acid, p-coumaric acid, monomethyl citrate, and protocatechuic acid. The retention time–molecular weight distribution showed that organic and phenolic acids were concentrated in the early chromatographic region, whereas most lignans and terpenoids eluted between approximately 15 and 22 min (Figure 1D). Characteristic SC lignans identified in the later-eluting region included Gomisin T, Schisandrin, Gomisin D, Gomisin J, Schisandrol B, Schisantherin B, Schisandrin B, Schisandrin C, Schisandrin A, Schisantherin A, Schisanhenol, Gomisin K1, and Gomisin M2. Ganwuweizic acid, Schisanlactone C, Kadsuphilactone B, β-sitosterol, and several Angeloylgomisin derivatives were also detected. These results demonstrate that the SC decoction has a complex chemical profile dominated by lignans and supplemented by organic acids, phenolic acids, terpenoids, and sterols.

### 3.2 SC alleviates DSS-induced UC and restores mucosal barrier integrity

**Figure 2:**
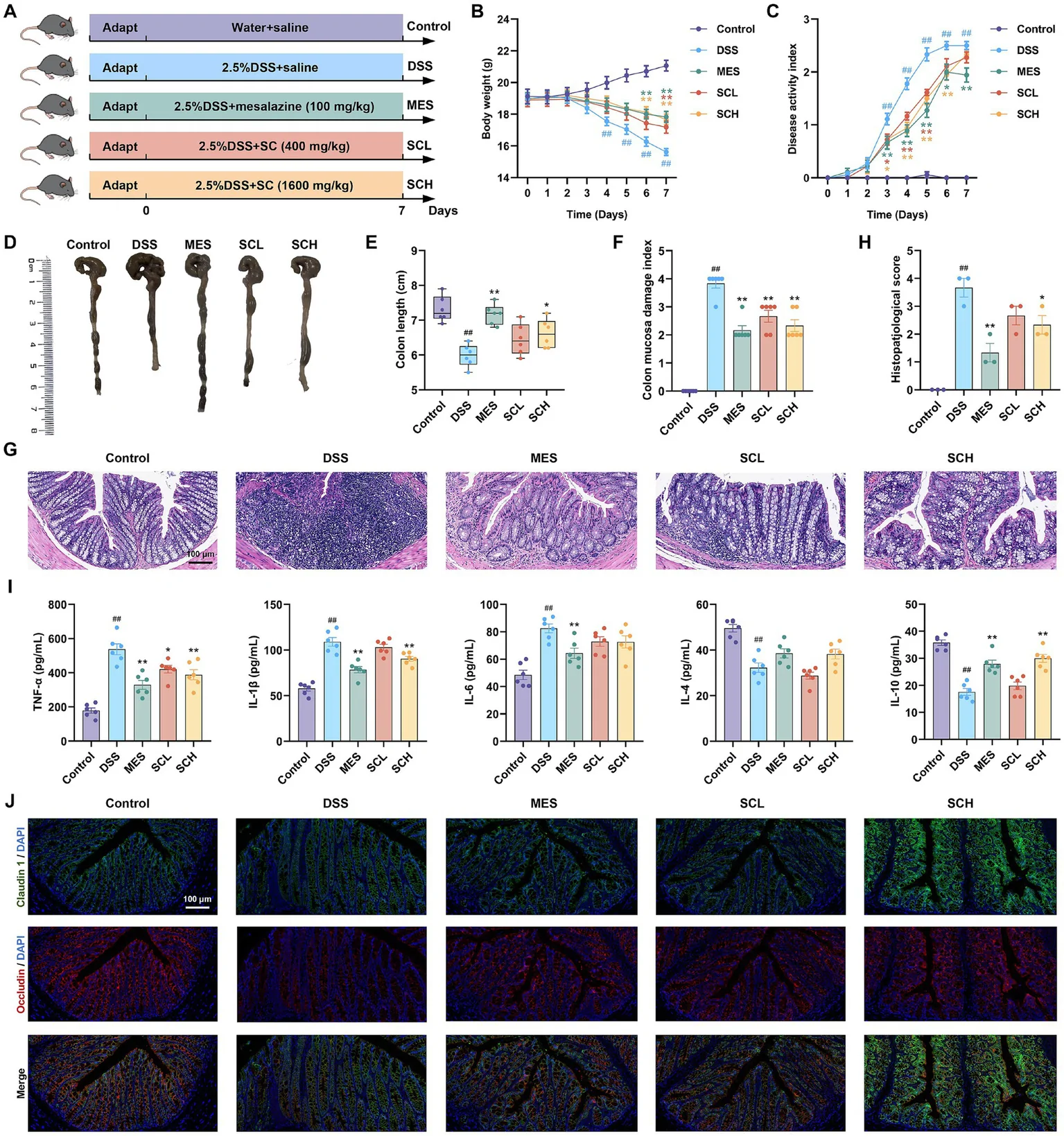
*Schisandra chinensis* alleviates DSS-induced colitis and restores intestinal barrier integrity. **(A)** Experimental design. **(B)** Body weight changes. **(C)** Disease activity index. **(D)** Representative colon images. **(E)** Colon length. **(F)** Colonic mucosal damage index. **(G)** Representative H&E-stained colon sections. **(H)** Histopathological scores. **(I)** Colonic levels of TNF-α, IL-1β, IL-6, IL-4, and IL-10. **(J)** Representative immunofluorescence images of claudin-1 and occludin. ^##^*p* < 0.01 versus the Control group; ^*^p < 0.05 and ^**^p < 0.01 versus the DSS group.

To evaluate the protective effect of SC against DSS-induced acute colitis, mice were assigned to Control, DSS, Mesalazine, SC low-dose, and SC high-dose groups and treated according to the protocol shown in Figure 2A. Body weight increased steadily and DAI scores remained low in Control mice. In contrast, DSS-treated mice began to lose substantial body weight during the middle and late stages of model induction. They also developed loose stools, fecal occult blood, and progressively increasing DAI scores, confirming successful induction of acute colitis (Figures 2B,C). Mesalazine and both doses of SC partially attenuated DSS-induced body weight loss and restrained the increase in DAI.

Gross examination of the colon supported these observations. DSS caused pronounced colon shortening, whereas mesalazine and SC restored colon length to varying degrees (Figures 2D,E). DSS also markedly increased the CMDI and histopathological score, both of which were reduced by SC treatment (Figures 2F,H). These findings indicate that SC attenuated systemic disease activity and macroscopic colonic injury in DSS-treated mice.

Hematoxylin and eosin (H&E) staining showed a continuous epithelium, regularly arranged crypts, a organized mucosal layer, and limited inflammatory cell infiltration in Control mice. In contrast, DSS caused extensive epithelial disruption, crypt loss or disorganization, mucosal and submucosal edema, and dense inflammatory cell infiltration. Mesalazine and SC improved epithelial continuity, preserved crypt architecture, and reduced the inflammatory infiltration and tissue damage (Figure 2G). These histological changes were consistent with the improvements in body weight, DAI, colon length, and CMDI.

The DSS substantially increased the colonic concentrations of the pro-inflammatory cytokines TNF-α, IL-1β, and IL-6 and reduced the anti-inflammatory cytokines IL-4 and IL-10, indicating marked local cytokine imbalance. SC reduced TNF-α, IL-1β, and IL-6 and restored IL-4 and IL-10 to varying degrees. Thus, SC suppressed DSS-induced pro-inflammatory responses and promoted partial recovery of mucosal immune homeostasis (Figure 2I).

Immunofluorescence was then used to assess epithelial tight junction proteins. Claudin-1 and occludin signals were strong and relatively continuous along the colonic epithelium in the Control group. DSS markedly reduced their fluorescence intensity and disrupted signal continuity. Mesalazine and SC restored both proteins to varying degrees, with a more evident improvement in the high-dose SC group (Figure 2J). Western blotting further confirmed that DSS reduced occludin and claudin-1 protein expression, whereas SC partially reversed these decreases (Supplementary Figure S1). Therefore, the protective effect of SC was associated not only with reduced inflammation, but also with preservation of tight junction proteins and repair of the intestinal mucosal barrier.

### 3.3 SC restructures the DSS-disrupted gut microbiome and enriches *Parabacteroides goldsteinii*

**Figure 3:**
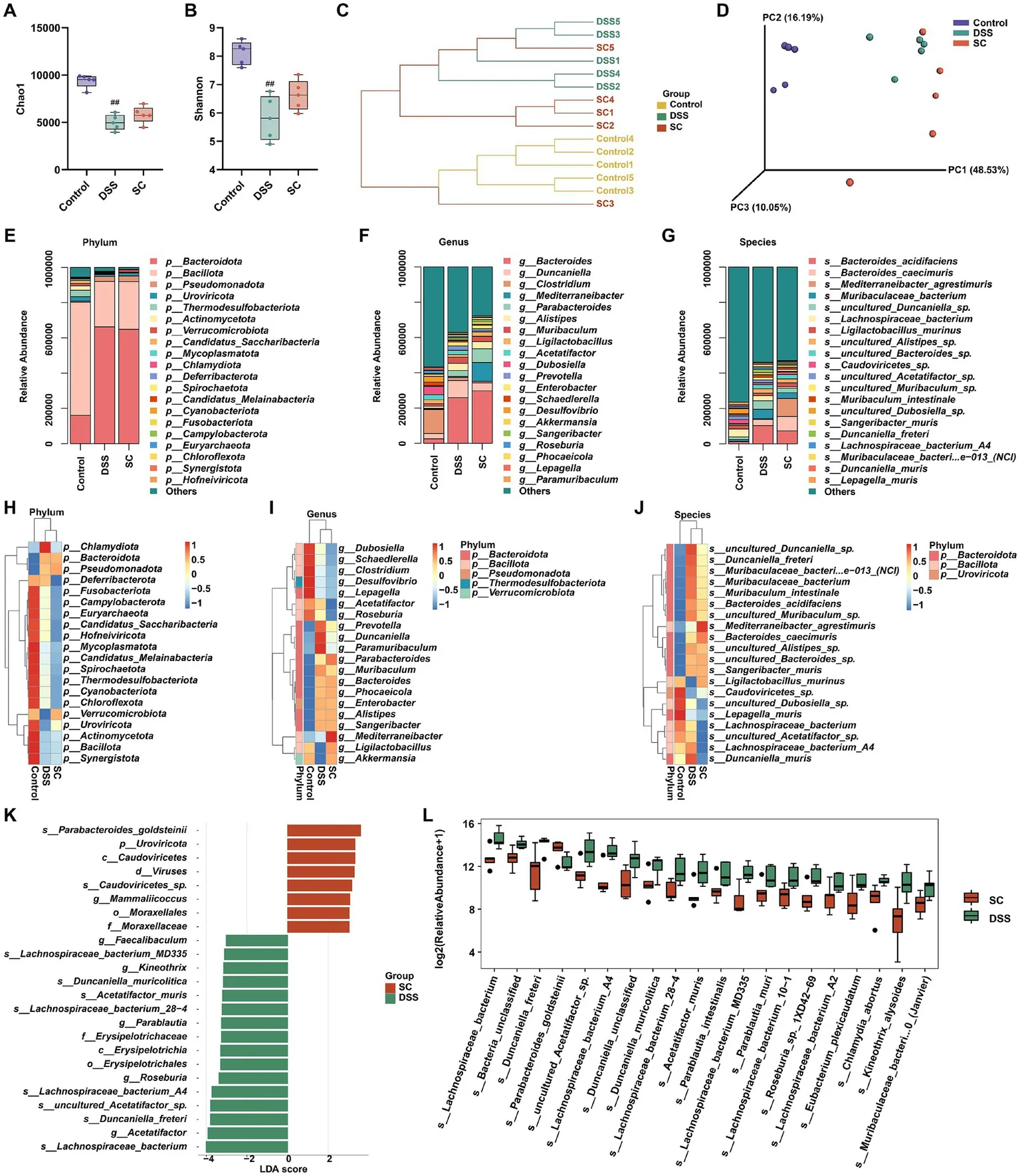
*Schisandra chinensis* restructures the DSS-disrupted gut microbiota and enriches *Parabacteroides goldsteinii*. **(A,B)** Chao1 and Shannon diversity indices. **(C)** Hierarchical clustering of microbial communities. **(D)** Principal coordinate analysis. **(E–G)** Relative abundances of dominant taxa at the phylum, genus, and species levels. **(H–J)** Heatmaps of representative bacterial taxa. **(K)** LEfSe analysis of taxa discriminating between the DSS and SC groups. **(L)** Relative abundances of representative differential species. ^##^*p* < 0.01 versus the control group.

To determine whether the colonic protection afforded by SC was associated with modulation of the gut microbiota, fecal samples from the Control, DSS, and SC groups were analyzed by metagenomic sequencing. Both the Chao1 and Shannon indices were lower in the DSS group than in the Control group, indicating reduced species richness and community diversity after DSS exposure. Both indices showed a recovery trend after SC treatment, suggesting partial restoration of the ecological complexity lost during colitis (Figures 3A,B).

β-diversity analysis further demonstrated clear differences in overall community structure among the three groups. Hierarchical clustering showed that most Control and DSS samples formed separate branches, whereas SC samples were clearly separated from the DSS samples and formed an independent cluster or occupied an intermediate position between the Control and DSS groups (Figure 3C). Principal coordinate analysis yielded a similar pattern. DSS shifted the microbial community away from the Control state, whereas SC induced a second restructuring of the microbiota (Figure 3D). These observations indicate that SC exerted a community-wide effect rather than altering only a few individual taxa.

At the taxonomic level, DSS markedly altered the relative abundance profiles at the phylum, genus, and species levels (Figures 3E–G). It changed the proportions of major phyla, including Bacteroidota and Bacillota, and affected several genera, including Bacteroides, Parabacteroides, Dubosiella, Ligilactobacillus, and Akkermansia. Species-level stacked bar plots and heatmaps showed that DSS induced broad alterations in microbial taxa. After SC treatment, some abundance patterns shifted toward those observed in the Control group, while others formed a distinct SC-associated ecological state (Figures 3H–J).

Linear discriminant analysis effect size (LEfSe) was used to identify taxa that discriminated between the DSS and SC groups. *P. goldsteinii* was among the species most strongly enriched in the SC group and made a major contribution to group separation (Figure 3K). Direct abundance comparisons confirmed that *P. goldsteinii* and several related species increased after SC treatment, whereas several Lachnospiraceae- and Acetatifactor-related taxa were relatively enriched in the DSS group (Figure 3L). On the basis of its abundance pattern and its relationship to the subsequent metabolic findings, *P. goldsteinii* was selected as a candidate functional bacterium that might contribute to SC-mediated protection.

Functional metagenomic analysis identified 6,517 core microbial genes shared among the Control, DSS, and SC groups, together with subsets of group-specific genes (Supplementary Figure S2A). Reporter-score analysis showed that, compared with the DSS group, the SC-associated microbiome was enriched in pathways including benzoate degradation, microbial metabolism in diverse environments, carotenoid biosynthesis, oxidative phosphorylation, degradation of aromatic compounds, and valine, leucine, and isoleucine degradation. In contrast, the DSS-associated microbiome was relatively enriched in O-antigen nucleotide-sugar biosynthesis, nucleotide biosynthesis, aminoacyl-tRNA biosynthesis, methane metabolism, and several functions related to bacterial structure and proliferation (Supplementary Figure S2B). The enhanced capacity for valine, leucine, and isoleucine degradation provided metagenomic evidence that SC modulated microbial BCAA metabolism.

### 3.4 SC remodels leucine/BCAA metabolism and promotes branched-chain fatty acid production

**Figure 4:**
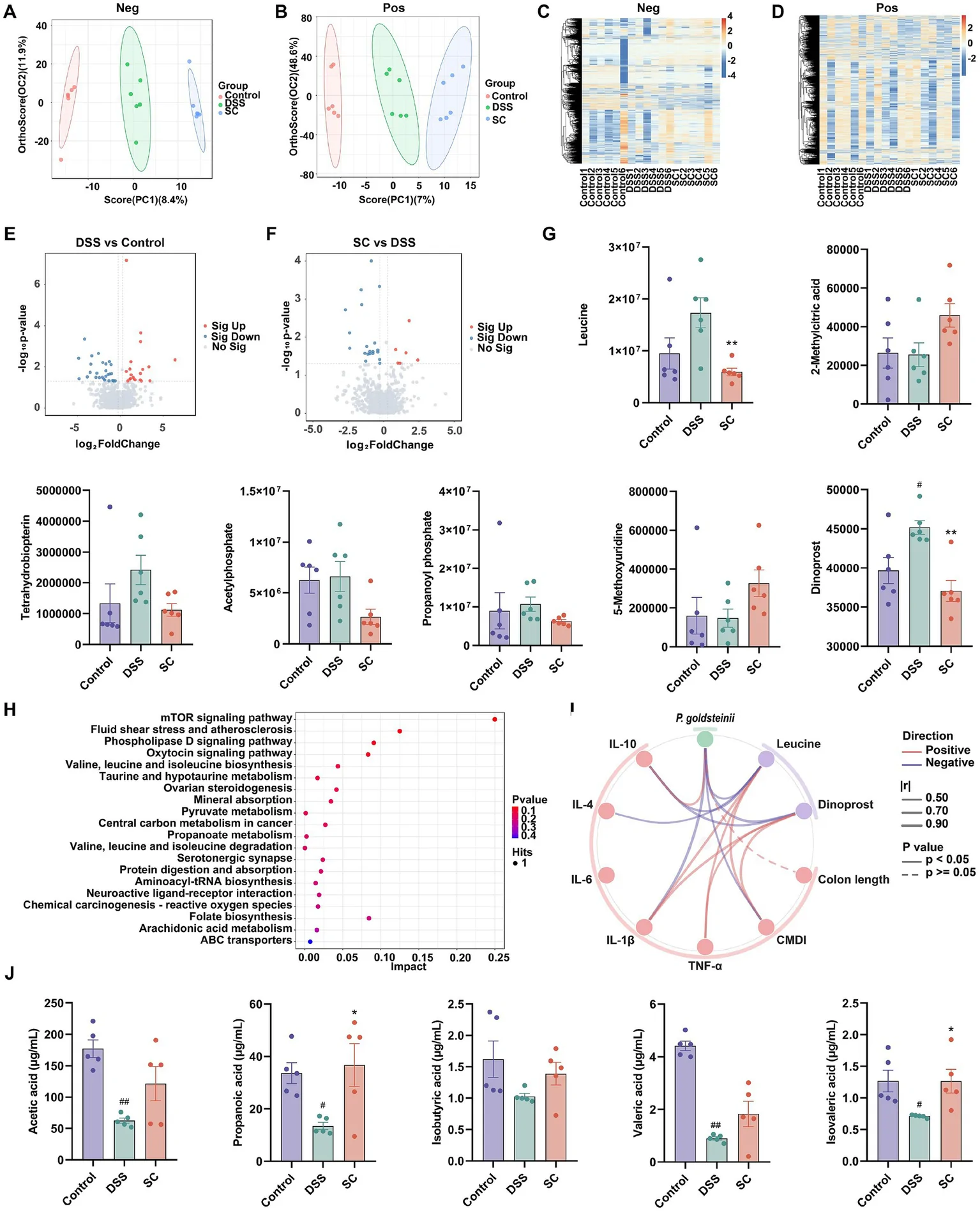
*Schisandra chinensis* remodels leucine/BCAA metabolism and promotes branched-chain fatty acid production. **(A,B)** OPLS-DA score plots in negative- and positive-ion modes. **(C,D)** Heatmaps of differential metabolites. **(E,F)** Volcano plots for the DSS-versus-Control and SC-versus-DSS comparisons. **(G)** Relative levels of representative differential metabolites. **(H)** Metabolic pathway enrichment analysis. **(I)** Correlation network linking *P. goldsteinii*, metabolites, inflammatory indices, and colon-injury parameters. **(J)** Fecal concentrations of acetic acid, propanoic acid, isobutyric acid, valeric acid, and isovaleric acid. ^#^*p* < 0.05 and ^##^*p* < 0.01 versus the control group; ^*^p < 0.05 and ^**^p < 0.01 versus the DSS group.

Untargeted metabolomics was performed to characterize the metabolic consequences of SC-mediated microbiota remodeling. Orthogonal partial least-squares discriminant analysis (OPLS-DA) in both positive- and negative-ion modes clearly separated the Control, DSS, and SC samples (Figures 4A,B). The metabolic profile of the DSS group deviated markedly from that of the Control group, while the SC group formed a separate cluster from the DSS group. These results indicate that DSS induced extensive metabolic disturbance and that SC substantially reshaped the fecal metabolome.

Heatmaps generated from both ionization modes showed that numerous metabolites were abnormally increased or decreased after DSS exposure. SC shifted a subset of these metabolites toward Control-like levels and generated additional metabolic features distinct from those of the DSS group (Figures 4C,D). Volcano plots identified multiple significantly altered metabolites in the DSS-versus-Control and SC-versus-DSS comparisons (Figures 4E,F), indicating that the metabolic response to SC involved several biological processes.

Among the representative differential metabolites, leucine was markedly increased in the DSS group and significantly decreased after SC treatment (Figure 4G). Leucine is not only a substrate for microbial fermentation, but also a major nutrient-sensing signal for mTORC1 (Liu and Sabatini, 2020; Wolfson et al., 2016). Its accumulation during DSS-induced colitis may therefore be associated with activation of inflammation-related nutrient signaling, whereas the decrease after SC treatment suggests enhanced utilization or degradation.

The inflammation-associated lipid mediator dinoprost was also increased in the DSS group and reduced by SC. In addition, SC increased 2-methylcitric acid and 5-methoxyuridine and tended to reduce tetrahydrobiopterin, acetyl phosphate, and propanoyl phosphate (Figure 4G). Thus, SC-mediated metabolic remodeling was not restricted to a single BCAA. It may also have affected propionyl-CoA-related metabolism, nucleotide metabolism, energy metabolism, and the production of arachidonic-acid-derived inflammatory mediators.

Pathway enrichment analysis indicated that differential metabolites were associated with mTOR signaling, valine/leucine/isoleucine biosynthesis, valine/leucine/isoleucine degradation, propanoate metabolism, pyruvate metabolism, central carbon metabolism, arachidonic-acid metabolism, aminoacyl-tRNA biosynthesis, and ABC transporters (Figure 4H). Concurrent enrichment of leucine-related pathways and mTOR signaling suggested that SC may regulate nutrient-sensitive mTOR activity by altering BCAA availability. Enrichment of BCAA degradation and propanoate metabolism was also consistent with microbial conversion of amino acid substrates into small organic acids.

Targeted analysis showed that DSS broadly reduced fecal acetate, propionate, isobutyrate, valerate, and isovalerate (Figure 4J). SC produced a pronounced recovery of propionate and isovalerate, while several other fatty acids showed more modest increases. Pairwise OPLS-DA based on the fatty acid profile clearly separated the Control and DSS groups and the DSS and SC groups (Supplementary Figure S3A). Butyrate and hexanoate were also reduced by DSS, although their recovery after SC treatment was limited (Supplementary Figure S3B). Because isovalerate showed a particularly sensitive response to SC, it was selected as a candidate microbial metabolite for functional validation.

A microbiota–metabolite–efficacy correlation network linked *P. goldsteinii*, leucine, and dinoprost to colon length, CMDI, TNF-α, IL-1β, IL-6, IL-4, and IL-10 (Figure 4I). These associations indicate that the candidate bacterium, leucine metabolism, and inflammatory phenotypes were embedded within a shared microbiota–metabolite–host network related to SC efficacy.

Taken together, the metagenomic and metabolomic data suggest that SC enriched functional bacteria, including *P. goldsteinii*, and enhanced BCAA degradation. These coordinated changes are consistent with enhanced microbial BCAA metabolism after SC treatment; however, direct conversion of leucine to isovalerate by *P. goldsteinii* was not demonstrated in the present study (Diether and Willing, 2019; Neis et al., 2015).

### 3.5 SC suppresses the mTOR/HIF-1α/STAT3/IL-17 inflammatory pathway in colonic tissue

**Figure 5:**
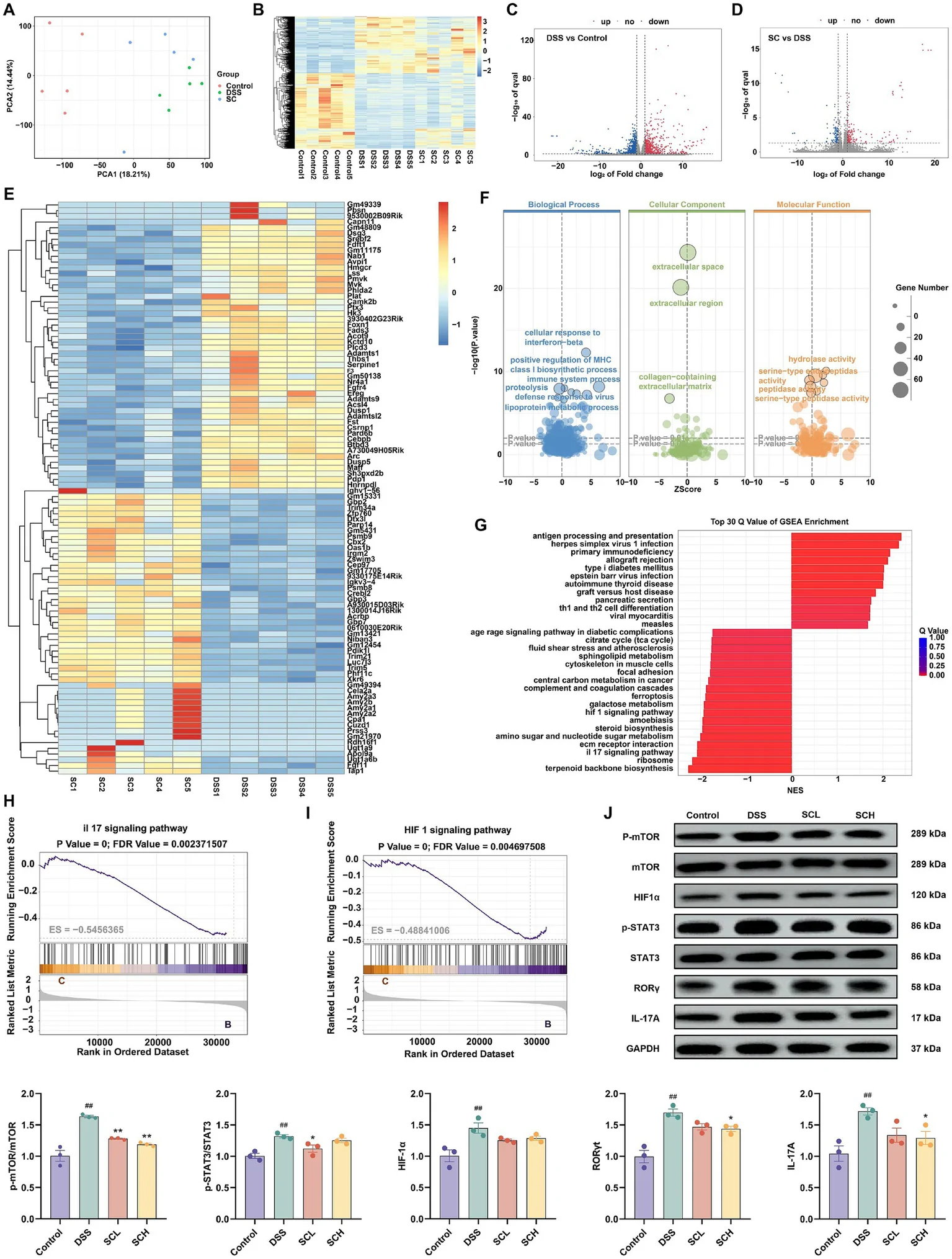
*Schisandra chinensis* suppresses the mTOR/HIF-1α/STAT3/IL-17 inflammatory pathway in colonic tissue. **(A)** PCA of colonic transcriptomes. **(B)** Heatmap of global gene-expression profiles across the control, DSS, and SC groups. **(C,D)** Volcano plots for the DSS-versus-control and SC-versus-DSS comparisons. **(E)** Heatmap of representative differentially expressed genes. **(F)** Gene Ontology enrichment analysis. **(G)** GSEA of representative pathways. **(H,I)** Enrichment plots for the IL-17 and HIF-1 signaling pathways. **(J)** Representative western blots and densitometric analysis of p-mTOR/mTOR, p-STAT3/STAT3, HIF-1α, RORγt, and IL-17A. ^##^*p* < 0.01 versus the ontrol group; ^*^p < 0.05 and ^**^p < 0.01 versus the cDSS group.

To define host molecular responses associated with the microbial and metabolic changes, colonic tissues from the Control, DSS, and SC groups were subjected to RNA sequencing. Principal component analysis (PCA) showed clear separation of the three groups, indicating that DSS induced extensive transcriptional changes and that SC substantially remodeled the DSS-associated colonic expression profile (Figure 5A). A global gene expression heatmap likewise revealed distinct expression patterns among the Control, DSS, and SC groups, with generally good within-group consistency (Figure 5B). Volcano plots for the DSS-versus-Control and SC-versus-DSS comparisons identified numerous differentially expressed genes (Figures 5C,D). A heatmap of representative genes further showed that SC reversed a subset of the expression abnormalities induced by DSS (Figure 5E). These results indicate that SC-mediated protection was accompanied by broad restructuring of transcriptional programs related to immunity, metabolism, and tissue repair.

Gene Ontology analysis showed that the differentially expressed genes were enriched in immune-response processes, including cellular response to interferon-β, positive regulation of MHC class I biosynthetic process, immune system process, and defense response to virus. Enriched cellular-component terms included extracellular space, extracellular region, and collagen-containing extracellular matrix, while molecular-function terms included hydrolase activity and serine-type peptidase activity (Figure 5F). These findings suggest that DSS-induced colitis involved not only abnormal mucosal immunity and antigen presentation, but also extracellular-matrix remodeling, altered protease activity, tissue damage, and repair. SC exerted coordinated effects on these processes.

Gene set enrichment analysis (GSEA) identified significant changes in antigen processing and presentation, infection-related immune pathways, Th1/Th2 cell differentiation, metabolic pathways, HIF-1 signaling, and IL-17 signaling (Figure 5G). In the SC-versus-DSS comparison, the HIF-1 and IL-17 signaling pathways were negatively enriched, suggesting that SC inhibited the excessive activation of HIF-1-associated inflammatory metabolism and IL-17-mediated mucosal inflammation induced by DSS (Figures 5H,I). Supplementary KEGG analysis likewise implicated PI3K–Akt signaling, NOD-like receptor signaling, HIF-1 signaling, antigen processing and presentation, and other inflammatory and metabolic pathways (Supplementary Figure S5).

Proteins associated with the mTOR/HIF-1α/STAT3/Th17–IL-17 axis were then assessed to validate the transcriptomic findings. Compared with the Control group, the DSS group showed increased p-mTOR/mTOR and p-STAT3/STAT3 ratios and higher HIF-1α, RORγt, and IL-17A protein levels (Figure 5J). SC reduced mTOR phosphorylation and markedly suppressed RORγt and IL-17A, while p-STAT3 and HIF-1α were also decreased to varying degrees. The high- and low-dose SC groups showed the same overall regulatory direction, although the magnitude of the effect varied among proteins and doses. To further validate the expression changes of key genes associated with this pathway, qRT-PCR was performed in colonic tissues. Compared with the Control group, DSS treatment significantly increased the mRNA levels of m-TOR, HIF-1α, RORC, and IL-17A (*p* < 0.01). SC treatment significantly reduced the DSS-induced increases in Hif1a, Rorc, and Il17a mRNA expression (*p* < 0.01), whereas Mtor mRNA expression was not significantly altered compared with the DSS group (Supplementary Figure S6). These results were generally consistent with the transcriptomic and protein-level findings and further supported the inhibitory effect of SC on HIF-1α/RORγt/IL-17-associated inflammatory signaling.

These findings connect the microbial and metabolic changes to host inflammatory signaling. SC reduced leucine, increased isovalerate, and suppressed nutrient-sensitive mTOR activity. This was accompanied by attenuation of HIF-1α- and STAT3-associated inflammatory programs and reduced RORγt and IL-17A expression.

### 3.6 The protective effect of SC is microbiota dependent and can be reproduced by *Parabacteroides goldsteinii*

**Figure 6:**
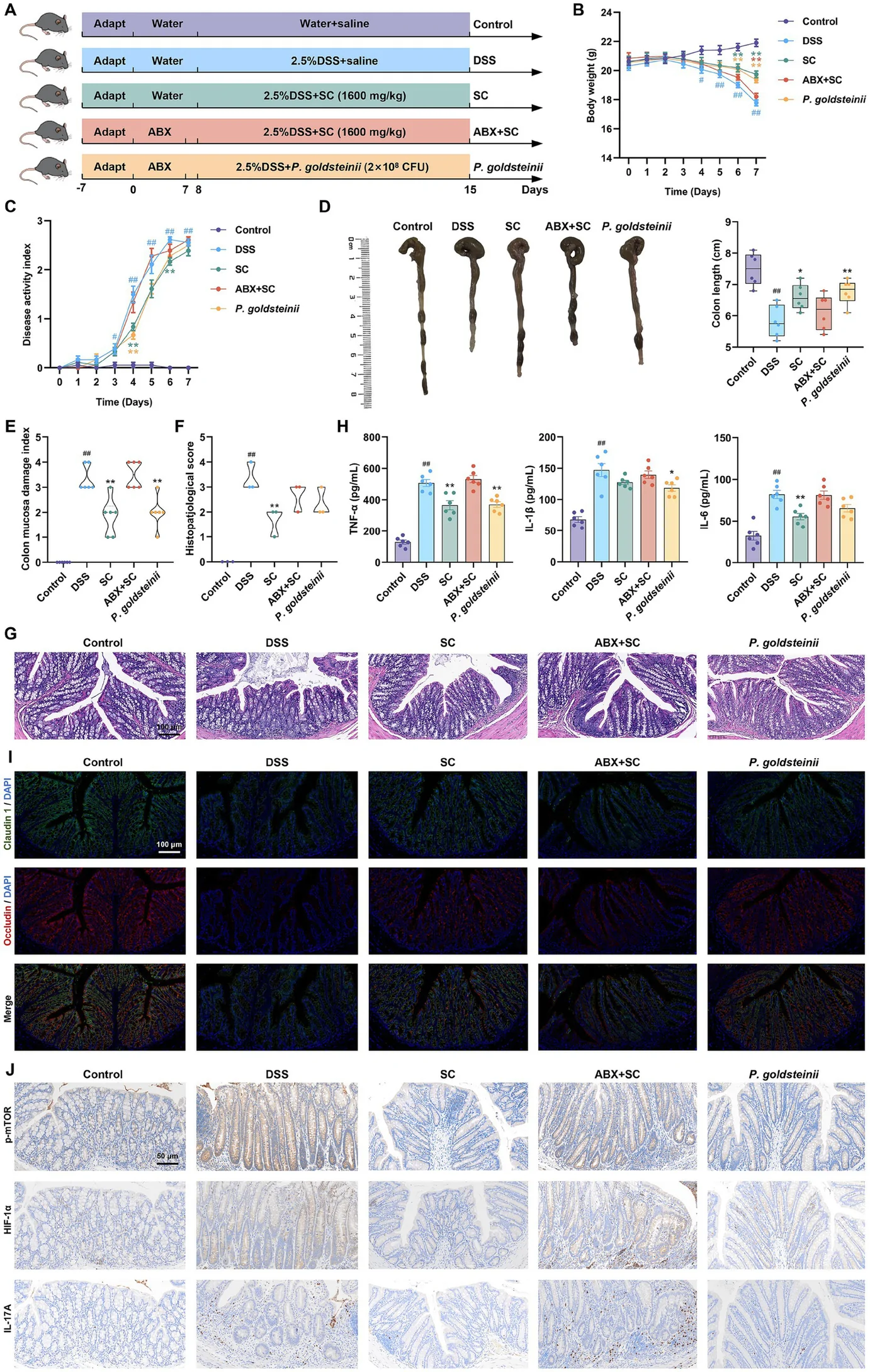
The protective effect of SC is microbiota dependent and can be reproduced by *P. goldsteinii*. **(A)** Experimental design for antibiotic treatment and *P. goldsteinii* supplementation. **(B)** Body weight changes. **(C)** Disease activity index. **(D)** Representative colon images and colon length. **(E)** Colonic mucosal damage index. **(F)** Histopathological scores. **(G)** Representative H&E-stained colon sections. **(H)** Colonic levels of TNF-α, IL-1β, and IL-6. **(I)** Representative immunofluorescence images of claudin-1 and occludin. **(J)** Representative immunohistochemical staining of p-mTOR, HIF-1α, and IL-17A. ^#^*p* < 0.05 and ^##^*p* < 0.01 versus the ontrol group; ^*^p < 0.05 and ^**^p < 0.01 versus the cDSS group.

To examine the functional contribution of the gut microbiota to SC efficacy, an antibiotic cocktail was used to deplete intestinal bacteria, and a separate group received *P. goldsteinii* supplementation (Figure 6A). As in the initial efficacy experiment, DSS caused progressive body weight loss, elevated DAI, colon shortening, and increased mucosal and histological injury (Figures 6B–F). SC significantly improved these disease indices. When SC was combined with antibiotic treatment, however, its protective effects were weakened. Compared with the SC group, mice in the ABX + SC group showed greater body weight loss, a trend toward higher DAI, shorter colons, and increased CMDI and histopathological scores (Figures 6B–F). These findings indicate that an intact or recoverable gut microbiota is important for the full protective effect of SC against colitis.

*Parabacteroides goldsteinii* supplementation reproduced part of the protective phenotype induced by SC. Compared with DSS-treated mice, mice receiving *P. goldsteinii* showed less body weight loss, lower DAI, longer colons, and reduced CMDI and histopathological scores (Figures 6B–F). H&E staining revealed extensive epithelial injury, crypt destruction, and inflammatory cell infiltration in the DSS group. SC and *P. goldsteinii* preserved epithelial and crypt architecture and reduced inflammatory infiltration, whereas histological protection was weaker in the ABX + SC group than in the SC group (Figure 6G).

The cytokine profile was consistent with the disease phenotype. SC significantly reduced the DSS-induced increases in TNF-α, IL-1β, and IL-6, whereas microbiota depletion partly weakened these effects (Figure 6H). *P. goldsteinii* supplementation also reduced all three pro-inflammatory cytokines. Supplementary analyses showed that DSS lowered IL-4 and IL-10. SC partially restored both cytokines, the recovery was attenuated by antibiotic treatment, and *P. goldsteinii* produced a partial increase (Supplementary Figure S4).

Immunofluorescence showed that DSS weakened and fragmented the epithelial claudin-1 and occludin signals. SC restored both the abundance and continuity of these tight junction proteins, whereas the recovery was weaker in the ABX + SC group. *P. goldsteinii* increased claudin-1 and occludin compared with DSS alone, indicating that this bacterium improved DSS-induced epithelial barrier injury (Figure 6I).

Immunohistochemistry further showed stronger p-mTOR, HIF-1α, and IL-17A staining in the DSS group. SC markedly reduced these signals, but its inhibitory effects were attenuated after microbiota depletion. *P. goldsteinii* supplementation also reduced p-mTOR, HIF-1α, and IL-17A staining (Figure 6J). Thus, *P. goldsteinii* was not merely a taxon associated with SC treatment. It may contribute to regulation of the mTOR/HIF-1α/IL-17 inflammatory pathway and intestinal barrier function.

Together, the antibiotic attenuation experiment and candidate-bacterium supplementation experiment indicate that the protective effect of SC against colitis is at least partly microbiota dependent. *P. goldsteinii* reproduced part of the anti-inflammatory, tissue-protective, and barrier-restorative effects of SC.

### 3.7 Sodium isovalerate mimics SC-mediated protection, whereas mTOR activation counteracts the effects of SC

**Figure 7:**
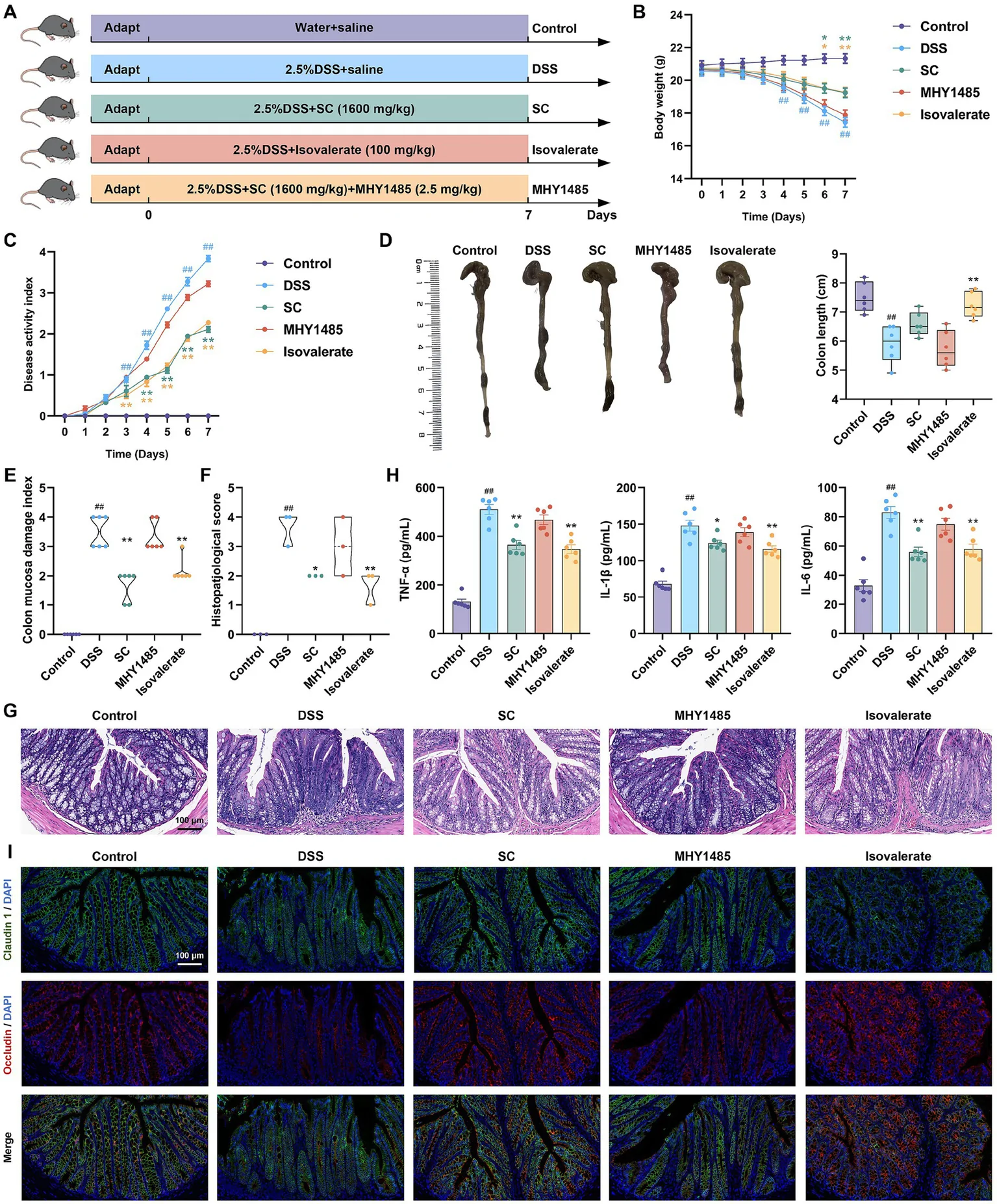
Sodium isovalerate mimics the protective effects of SC, whereas mTOR activation counteracts SC-mediated protection. **(A)** Experimental design for sodium isovalerate supplementation and MHY1485 treatment. **(B)** Body weight changes. **(C)** Disease activity index. **(D)** Representative colon images and colon length. **(E)** Colonic mucosal damage index. **(F)** Histopathological scores. **(G)** Representative H&E-stained colon sections. **(H)** Colonic levels of TNF-α, IL-1β, and IL-6. **(I)** Representative immunofluorescence images of claudin-1 and occludin. ^##^*p* < 0.01 versus the Control group; ^*^p < 0.05 and ^**^p < 0.01 versus the DSS group.

To assess the functional relevance of isovalerate and mTOR during SC treatment, mice were assigned to Control, DSS, SC, sodium isovalerate, and SC + MHY1485 groups. MHY1485 was used as an mTOR activator to determine whether pharmacological mTOR activation weakened the therapeutic effects of SC (Figure 7A).

DSS again caused progressive body weight loss and increased DAI, both of which were attenuated by SC (Figures 7B,C). Sodium isovalerate similarly reduced body weight loss and DAI, producing an overall improvement comparable in direction to that of SC. In contrast, the combination of SC and MHY1485 provided less protection than SC alone, as shown by greater body weight loss and increased disease activity. These results support isovalerate as a functional metabolite contributing to SC-mediated protection and indicate that mTOR activation counteracts part of the effect of SC.

Gross colonic morphology and tissue injury indices supported this interpretation. SC and sodium isovalerate attenuated DSS-induced colon shortening and reduced both CMDI and histopathological scores. Compared with SC alone, the SC + MHY1485 group had shorter colons and higher mucosal-injury and histopathological scores (Figures 7D–F). H&E staining showed severe epithelial disruption, crypt injury, and inflammatory cell infiltration in the DSS group. SC and sodium isovalerate improved mucosal architecture, preserved more intact crypts, and reduced inflammatory infiltration, whereas MHY1485 weakened the histological protection provided by SC (Figure 7G).

The SC and sodium isovalerate also reduced the DSS-induced increases in TNF-α, IL-1β, and IL-6. Addition of MHY1485 to SC led to partial recovery of these pro-inflammatory cytokines relative to SC alone (Figure 7H). Thus, sodium isovalerate reproduced part of the anti-inflammatory effect of SC, whereas sustained mTOR activation was unfavorable for suppression of colonic inflammation.

Immunofluorescence showed that both SC and sodium isovalerate restored claudin-1 and occludin signals and improved their continuous distribution along the colonic epithelium. The fluorescence intensity and continuity of both proteins were lower in the SC + MHY1485 group than in the SC group, indicating that mTOR activation impaired the barrier-protective effect of SC (Figure 7I). The sodium isovalerate group showed a particularly evident recovery of occludin, further supporting a direct protective effect of isovalerate on the epithelial barrier.

In summary, sodium isovalerate partly mimicked the anti-inflammatory, tissue-protective, and barrier-restorative effects of SC in DSS-induced colitis, whereas pharmacological activation of mTOR reduced SC efficacy. Together with the metagenomic and metabolomic findings, these results support an association among SC-induced microbiota remodeling, altered leucine/isovalerate metabolism, and attenuation of mTOR/HIF-1α/IL-17 signaling. Direct causal relationships among these events remain to be established.

## 4 Discussion

This study systematically characterized the protective effects of SC against DSS-induced colitis at the levels of chemical composition, overall efficacy, gut microbial ecology, microbial metabolism, and host inflammatory signaling. SC attenuated body weight loss, disease activity, colon shortening, mucosal injury, and inflammatory cell infiltration and restored claudin-1 and occludin expression. Multi-omics analyses further indicated that SC enriched *P. goldsteinii*, enhanced microbial BCAA-degradation functions, reduced the abnormally elevated level of leucine, increased isovalerate, and suppressed the colonic mTOR/HIF-1α/STAT3/IL-17 inflammatory program. Microbiota depletion weakened SC efficacy, while *P. goldsteinii* and sodium isovalerate reproduced part of the protective phenotype. Conversely, MHY1485 reduced the benefits of SC. Together, these findings support a model in which microbiota-dependent metabolic remodeling links SC treatment to host inflammatory signaling.

UPLC-Q-TOF-MS/MS showed that the SC decoction was dominated by lignans and also contained organic acids, phenolic acids, terpenoids, and sterols. SC lignans possess anti-inflammatory and antioxidant activities, whereas phenolic acids, organic acids, and water-soluble macromolecules may be more likely to remain in the intestinal lumen and interact with the microbiota (Rybnikář et al., 2024; Skalski et al., 2025). A traditional decoction is not a single-compound system, and its protective activity against colitis may therefore involve both direct host effects and microbiota-mediated actions. Rather than attributing efficacy to a single lignan, we used chemical profiling to define the material basis of the decoction and improve experimental reproducibility. Previous studies showed that SC extracts and polysaccharides reduce DSS-induced cytokine production, restore tight junction proteins, and modify the gut microbiota. The basic pharmacological findings of the present study are consistent with those reports (Bian et al., 2022; Guo et al., 2024; Su et al., 2020; Wang et al., 2023).

A major finding was that SC extensively restructured the DSS-disrupted microbiota and enriched *P. goldsteinii* as a representative SC-associated species. Interest in *P. goldsteinii* initially arose from evidence that it contributed to the anti-obesity effects of fungal polysaccharides. Subsequent studies suggested that it can improve metabolism, strengthen barrier function, and suppress inflammation (Wu et al., 2019). More recently, Pericarpium Citri Reticulatae polysaccharides were shown to enrich *P. goldsteinii* and alleviate DSS-induced colitis by inhibiting LPS-associated PI3K–Akt signaling. Other studies implicated bile acid metabolism or valine–isobutyrate metabolism in the anti-colitis effects of this bacterium (He et al., 2025; Li et al., 2024; Qin et al., 2025). These findings suggest that *P. goldsteinii* does not act through one fixed host pathway. Its functional output is likely shaped by strain identity, available substrates, and the dietary environment. The present study extends this functional framework by associating *P. goldsteinii* enrichment with SC-induced remodeling of leucine and BCAA metabolism.

Antibiotic treatment attenuated the beneficial effects of SC on disease activity, tissue injury, inflammatory cytokines, and tight junction proteins, indicating that the gut microbiota contributes substantially to SC efficacy. Supplementation with *P. goldsteinii* alone improved DSS-induced colonic injury and reduced p-mTOR, HIF-1α, and IL-17A, thereby providing functional support for the metagenomic screening results. Nevertheless, the ABX + SC group retained partial protection, demonstrating that SC was not completely microbiota dependent. Lignans, organic acids, and other small molecules in the decoction may be absorbed by the host and act directly on epithelial or immune cells. Broad-spectrum antibiotics also alter bile acid pools, short-chain fatty acids, the mucus layer, and basal immune status. Therefore, attenuation of efficacy by antibiotics demonstrates participation of the microbiota as a whole but does not establish any single species as the exclusive mediator (Kennedy et al., 2018; Morgun et al., 2015).

*Schisandra chinensis* enhanced the microbial valine, leucine, and isoleucine degradation pathway, reduced leucine, and increased fecal isovalerate. Leucine is one of the most potent nutrient signals for mTORC1 and promotes its lysosomal recruitment and activation through the Sestrin2–GATOR2–Rag GTPase system (Liu and Sabatini, 2020; Saxton et al., 2016; Wolfson et al., 2016). The concurrent increases in leucine and p-mTOR in DSS-treated mice are therefore biologically concordant. Their simultaneous reduction after SC treatment suggests that limiting excessive leucine signaling may contribute to mTOR inhibition. Intestinal bacteria degrade BCAAs to generate isobutyrate, 2-methylbutyrate, and isovalerate, with isovalerate derived mainly from leucine fermentation (Diether and Willing, 2019; Neis et al., 2015; Portune et al., 2016). Recent work showed that isovalerate increases transepithelial electrical resistance, reduces paracellular permeability, and enhances epithelial barrier function partly through histone deacetylase inhibition (Beaumont et al., 2026). This evidence is consistent with the restoration of claudin-1 and occludin and the amelioration of colitis observed after sodium isovalerate administration in the present study.

Current evidence, however, is insufficient to conclude that SC-enriched *P. goldsteinii* directly converts leucine to isovalerate. Functional enrichment of the metagenome, the correlation network, reduced leucine, increased isovalerate, and bacterial supplementation together establish a coherent association and functional sequence, but direct strain-level metabolic evidence is lacking. This distinction is particularly important because a recent *P. goldsteinii* study identified a valine–isobutyrate pathway, whereas the present study proposes a leucine–isovalerate pathway. Although both belong to BCAA catabolism, they cannot be treated as equivalent (He et al., 2025). Future work should culture *P. goldsteinii* anaerobically with leucine as the substrate and directly quantify isovalerate in the culture supernatant. Stable-isotope tracing with ^13^C₆-leucine should be used to detect ^13^C-labeled isovalerate. Comparing the effects of the complete SC decoction, a polysaccharide-depleted fraction, and a lignan fraction on bacterial growth and metabolic output would also help identify the SC constituents that drive this process.

Transcriptomic and protein analyses showed that SC suppressed HIF-1- and IL-17-associated transcriptional programs and reduced p-mTOR, p-STAT3, HIF-1α, RORγt, and IL-17A. In addition to controlling cell growth and autophagy, mTOR contributes to the metabolic programming of CD4 + T-cell effector lineages (Liu and Sabatini, 2020). HIF-1α enhances glycolysis, promotes RORγt-dependent Th17 differentiation, and regulates the Th17/Treg balance (Dang et al., 2011; Shi et al., 2011). STAT3 is a major transcriptional mediator through which cytokines such as IL-6 induce RORγt and IL-17 expression (Kumar et al., 2021; Stockinger and Omenetti, 2017). The coordinated changes in mTOR, HIF-1α, STAT3, RORγt, and IL-17A observed here are therefore biologically plausible. MHY1485 weakened the effects of SC on body weight, DAI, colon length, inflammatory cytokines, and barrier proteins, further supporting the involvement of mTOR activation in SC efficacy. However, MHY1485 is not tissue- or cell-type-specific and can affect autophagy and multiple downstream mTOR processes. The data therefore support the conclusion that mTOR activation counteracted SC-mediated protection, but they do not establish that SC acts exclusively or specifically through mTOR (Choi et al., 2012).

*Schisandra chinensis* also reduced dinoprost and altered propanoate metabolism, propionyl-CoA-related intermediates, and several energy metabolism pathways. Thus, the metabolic effects of SC were unlikely to be restricted to the leucine–isovalerate axis. Microbial dysfunction in UC commonly involves amino acid, lipid, bile acid, and carbohydrate metabolism simultaneously (Franzosa et al., 2019; Lavelle and Sokol, 2020; Lloyd-Price et al., 2019; Ning et al., 2023). Leucine and isovalerate should therefore be regarded as key nodes that received substantial functional validation in the present multi-omics network, rather than as the sole explanation for all SC activity. Integration of additional differential metabolites with microbial genes, host receptors, and specific cell populations may reveal complementary mechanisms.

This study has several strengths. First, chemical profiling defined the composition of the SC decoction. Second, metagenomics, metabolomics, and transcriptomics provided a continuous line of evidence from microbial function to host signaling. Third, microbiota depletion, candidate-bacterium supplementation, metabolite replacement, and pharmacological mTOR activation were used as layered functional tests, moving the study beyond a purely correlative multi-omics analysis. Finally, the barrier-protective effect of isovalerate, a comparatively neglected branched-chain fatty acid, was supported *in vivo* and was consistent with recent evidence from intestinal organoid-derived epithelial monolayers (Beaumont et al., 2026).

Several limitations should also be acknowledged. First, the DSS model primarily reflects acute epithelial injury and innate immune activation and does not fully reproduce the chronic relapsing course or complex immune background of human UC (Wirtz et al., 2017; Yang and Merlin, 2024). Second, the relationships among *P. goldsteinii*, leucine, isovalerate, and p-mTOR/IL-17 signaling have not been validated in patients with UC. Third, direct production of isovalerate by *P. goldsteinii* was not demonstrated using pure culture, metatranscriptomics, strain-level gene disruption, or stable-isotope tracing. Fourth, the cellular sources of the altered mTOR and STAT3 signaling were not resolved. Because these proteins were assessed in whole-colon tissues, the observed changes may have originated from intestinal epithelial cells, macrophages, Th17 cells, or multiple cell populations. Future studies using flow cytometry and cell-specific analyses are required to clarify the principal responding cell types. Fifth, microbiota depletion and MHY1485 treatment can produce nonspecific effects. Future studies should employ germ-free or defined-microbiota mice, a controlled *P. goldsteinii* colonization model, colonic organoids, flow-cytometric analysis of CD4 + T-cell populations, and cell-specific manipulation of mTOR signaling.

In conclusion, SC remodeled the DSS-disrupted intestinal ecosystem and BCAA metabolic environment, enhanced *P. goldsteinii*-associated metabolic functions, reduced leucine, and increased isovalerate. These changes were accompanied by suppression of the mTOR/HIF-1α/STAT3/IL-17 inflammatory program and improvement of the intestinal mucosal barrier and colonic inflammation. The study proposes a mechanistic framework connecting a botanical medicine, a candidate functional bacterium, an amino acid-derived microbial metabolite, and host nutrient-sensing signaling. It also provides a basis for further investigation of SC and microbiota-derived isovalerate as interventions for intestinal inflammation.

## Data Availability

The metabolomics data generated in this study have been deposited in the MetaboLights repository (accession number: MTBLS15496).
