# Supplementary material for: Schisandra chinensis alleviates dextran sulfate sodium-induced colitis in association with remodeling of a Parabacteroides goldsteinii-related leucine–isovalerate metabolic axis and attenuation of mTOR/HIF-1α/IL-17 signaling
Source: Front Microbiol. 2026 Sep 9;17:1941347. doi: 10.3389/fmicb.2026.1941347 (PMC13597817; doi:10.3389/fmicb.2026.1941347)
Supplement: Supplementary file 1 [file Supplementary_file_1.docx]

Supplementary Table S1. Chemical constituents tentatively identified in the Schisandra chinensis by UPLC-Q-TOF-MS/MS.

| No. | Retention time | Molecular formula | Ion mode | Molecular weight‌ | ppm | m/z | Compounds |
| --- | --- | --- | --- | --- | --- | --- | --- |
| 1 | 0.74 | C_9_H_10_O_5_ | [M+H]^+^ | 199.0601 | 1.7 | / | Syringic acid |
| 2 | 0.83 | C_7_H_6_O_3_ | [M+H]^+^ | 139.0389 | -3.6 | / | 4-Hydroxybenzoic acid |
| 3 | 1.03 | C_16_H_18_O_9_ | [M+H]^+^ | 355.1023 | -0.8 | / | Chlorogenic acid |
| 4 | 1.14 | C_6_H_8_O_7_ | [M+H]^+^ | 193.0342 | -0.7 | 68.9977, 111.0083, 139.0031 | Citric acid |
| 5 | 1.44 | C_9_H_8_O_3_ | [M+H]^+^ | 165.0546 | -0.1 | 139.0002, 121.9680, 113.9630, 91.0519, 72.9359, 68.9949 | p-Coumaric acid |
| 6 | 2.8 | C_7_H_10_O_7_ | [M+H]^+^ | 207.0497 | -0.9 | 68.9974, 139.0024, 143.0325 | Monomethyl citrate |
| 7 | 3.23 | C_7_H_6_O_4_ | [M+H]^+^ | 155.0332 | -4 | 240.7599, 238.3618, 81.0697, 72.9367, 67.9358, 65.0377 | Protocatechuic acid |
| 8 | 9.46 | C_30_H_46_O_3_ | [M+H]^+^ | 455.3489 | -6.7 | 437.3615, 455.3461 | Ganwuweizic acid |
| 9 | 13.82 | C_15_H_22_O | [M+H]^+^ | 219.1744 | 0.6 | 79.0528, 133.1002, 203.1428, 219.1734 | Chamigrenal |
| 10 | 15.48 | C_29_H_38_O_9_ | [M+H]^+^ | 531.2588 | -3.4 | 531.1568, 453.2366, 401.1460, 341.0978, 191.1238 | Angeloylgomisin Q |
| 11 | 15.92 | C_23_H_30_O_7_ | [M+H]^+^ | 419.2064 | -0.1 | 149.0229, 149.1191, 195.0698, 313.1114, 338.1530, 323.1296, 369.1725, 370.1811, 401.1967, 419.1645, 419.2563 | Gomisin T |
| 12 | 16.69 | C_24_H_32_O_7_ | [M+H]^+^ | 433.2222 | 0.3 | 307.1298, 323.1269, 328.1298, 331.1171, 338.1500, 346.1408, 369.1685, 384.1931, 385.1678, 386.1713, 400.1879, 415.2117 | Schisandrin |
| 13 | 16.7 | C_29_H_50_O | [M+H]^+^ | 415.3906 | -5.3 | / | beta-Sitosterol |
| 14 | 16.7 | C_30_H_40_O_5_ | [M+H]^+^ | 481.2948 | -2.8 | / | Schisanlactone C |
| 15 | 16.7 | C_29_H_28_O_9_ | [M+H]^+^ | 521.1806 | -8.6 | 539.1533, 521.1795, 461.1736, 369.1617, 332.1339 | Schisantherin D |
| 16 | 16.92 | C_28_H_34_O_10_ | [M+H]^+^ | 531.2221 | -0.6 | 175.0773, 313.1045, 321.1068, 341.0990, 353.1365, 383.1464, 401.1578, 485.2121, 531.2225 | Gomisin D |
| 17 | 17.04 | C_22_H_28_O_6_ | [M+H]^+^ | 389.1961 | 0.8 | 255.0654, 287.0926, 319.1183, 357.1702, 374.1742, 389.1970 | Gomisin J |
| 18 | 17.24 | C_23_H_28_O_7_ | [M+H]^+^ | 417.1907 | -0.1 | 221.0582, 255.0658, 284.0679, 315.1178, 314.1096, 330.1095, 399.1767, 399.2147, 417.2092, 417.2472 | Schisandrol B |
| 19 | 17.26 | C_22_H_38_O_10_ | [M+H]^+^ | 463.2533 | -0.9 | 147.0809, 287.0465, 300.0971, 369.1645, 399.1785, 400.1787, 463.2639 | Anatolioside |
| 20 | 17.53 | C_28_H_34_O_9_ | [M+H]^+^ | 515.2280 | 1 | 113.0608, 285.0735, 298.0835, 343.1199, 355.1553, 385.1673, 515.2306 | Schisantherin B |
| 21 | 17.59 | C_22_H_30_O_6_ | [M+H]^+^ | 391.2117 | 0.6 | 163.0761, 167.0713, 205.1238, 237.1515, 391.2133 | Pregomisin |
| 22 | 17.97 | C_23_H_28_O_6_ | [M+H]^+^ | 401.1962 | 0.9 | 227.0703, 281.0816, 299.0929, 295.1331, 318.1117, 337.1447, 323.1293, 370.1797, 386.1736, 401.1980 | Schisandrin B |
| 23 | 17.97 | C_28_H_34_O_7_ | [M+H]^+^ | 483.2381 | 0.9 | 55.0541, 83.0489, 219.1012, 262.1201, 379.1176, 395.1481, 483.2367 | Gedunin |
| 24 | 17.97 | C_28_H_36_O_8_ | [M+H]^+^ | 501.2487 | 1 | 83.0492, 291.1011, 309.1479, 327.1223, 337.1422, 369.1679, 370.1757, 401.1944, 483.2376 | Angeloylgomisin H |
| 25 | 18.07 | C_22_H_24_O_6_ | [M+H]^+^ | 385.1649 | -4.7 | 316.0866, 355.1455, 385.1562, 301.0637, 298.07449 | Schisandrin C |
| 26 | 18.2 | C_30_H_42_O_5_ | [M+H]^+^ | 483.3105 | -0.1 | 483.2303, 468.2047, 452.2090, 414.1608, 381.1256 | Kadsuphilactone B |
| 27 | 18.24 | C_24_H_30_O_7_ | [M+H]^+^ | 431.2066 | 0.6 | 271.0962, 298.1201, 342.1483, 357.1444, 358.1436, 341.1398, 356.1635, 387.1830, 416.1851, 431.2090 | Schinsanlignone A |
| 28 | 18.24 | C_31_H_36_O_9_ | [M+H]^+^ | 553.2429 | -0.5 | 553.2327, 453.1819, 431.1993, 387.1739, 356.1541, 341.1288 | Benzoylgomisin Q |
| 29 | 18.63 | C_23_H_30_O_6_ | [M+H]^+^ | 403.2120 | 1.2 | 199.0753, 241.0860, 270.0895, 271.0963, 287.0922, 301.1080, 302.1160, 333.1334, 388.1886, 403.2111 | Schisanhenol |
| 30 | 18.64 | C_23_H_30_O_6_ | [M+H]^+^ | 403.2115 | -2.5 | 403.2049, 388.1823, 302.1095, 301.1018, 287.0869 | Gomisin K1 |
| 31 | 18.79 | C_30_H_32_O_9_ | [M+H]^+^ | 537.2115 | -0.7 | 537.2106, 437.1583, 415.1760, 371.1500, 340.1316 | Schisantherin A |
| 32 | 19.56 | C_22_H_26_O_6_ | [M+H]^+^ | 387.1802 | -2.8 | 387.1752, 372.1510, 287.0871, 227.0653, 199.0715 | Gomisin M2 |
| 33 | 19.91 | C_28_H_36_O_6_ | [M-H]^-^ | 467.2438 | -0.1 | / | Angeloylgomisin M1 |
| 34 | 20.19 | C_20_H_26_O_4_ | [M-H]- | 329.1768 | 3.1 | 99.0840, 127.1195, 146.9232, 155.1111, 181.1231, 199.1322, 201.1128, 197.1221, 243.1516, 271.1370, 271.1517, 299.1321, 329.2350, 329.1770, 329.1650 | Dihydroguaiaretic acid |
| 35 | 20.23 | C_24_H_32_O_6_ | [M+H]^+^ | 417.2277 | 1.4 | 241.0852, 315.1223, 285.1103, 301.1062, 316.1299, 402.2030, 417.2263 | Schisandrin A |
| 36 | 20.84 | C_15_H_24_ | [M+H]^+^ | 205.1950 | -0.2 | 67.0546, 95.0850, 149.0235, 149.1311, 205.1983 | beta-Elemene |
| 37 | 21.16 | C_28_H_36_O_7_ | [M+H]^+^ | 485.2535 | 0.4 | 55.0540, 83.0486, 403.2088, 485.2546 | Angeloylgomisin K3 |
| 38 | 21.46 | C_30_H_32_O_8_ | [M+H]^+^ | 521.2166 | -2.4 | 521.2522, 424.0839, 399.1737, 369.1655, 330.1083 | Benzoylisogomisin O |

**Supplementary Figure S1.** SC restores tight-junction protein expression in DSS-induced colitis. (A) Representative western blots of Occludin and Claudin-1 in colonic tissues. (B) Densitometric analysis of Occludin and Claudin-1 expression. ^##^*p* < 0.01 versus control group; ^*^*p* < 0.05 versus DSS group.

**Supplementary Figure S2.** SC alters the microbial gene repertoire and predicted metabolic functions. (A) Venn diagram showing shared and group-specific microbial genes among the Control, DSS, and SC groups. (B) Reporter-score analysis of differential microbial pathways between the DSS and SC groups.

**Supplementary Figure S3.** SC partially restores the fecal fatty-acid profile disrupted by DSS. (A) OPLS-DA score plots based on fecal fatty-acid profiles. (B) Fecal concentrations of butyric acid and caproic acid. ^##^*p* < 0.01 versus control group.

**Supplementary Figure S4.** Effects of microbiota depletion and P. goldsteinii supplementation on anti-inflammatory cytokines. Colonic levels of IL-4 (A) and IL-10 (B) in the indicated groups. ^##^*p* < 0.01 versus control group; ^*^*p* < 0.05, ^**^*p* < 0.01 versus DSS group.

**Supplementary Figure S5.** KEGG pathway distribution of differentially expressed genes after SC treatment.
